# Effect of FXR agonist GW4064 in the treatment of hilar cholangiocarcinoma in rats

**DOI:** 10.1038/s41598-022-23539-5

**Published:** 2022-11-07

**Authors:** Jie-ping Wang, Meng-yu Zhang, Ming Luo, Shu Qin, Xian-ming Xia

**Affiliations:** 1 Department of Rehabilitation, The Affiliated Hospital of Southwest Medical University, Luzhou, 646000 China; 2 Department of General Surgery (Hepatobiliary Surgery), The Affiliated Hospital of Southwest Medical University, Luzhou, 646000 Sichuan Province China

**Keywords:** Cancer, Cancer prevention

## Abstract

The study objective was to observe the treatment effect of the farnesoid X receptor (FXR) agonist GW4064 in a rat model of hilar cholangiocarcinoma to explore a new therapeutic target for gene therapy for hilar cholangiocarcinoma. Eighty male Wistar rats were randomly divided into four groups (treatment group, model group, control group and sham operation group, 20 rats in each group). The four groups were fed a standard diet. The treatment group and the model group were injected with a suspension of cholangiocarcinoma QBC939 cells into the hilar bile duct with a microsyringe, the control group was injected with normal saline, and the sham operation group was not injected with anything. A modified tail suspension test (TST) was used to evaluate the vitality of the rats. At 4 weeks, one rat in the treatment group and model group was euthanized, and the changes in the hilar bile duct were recorded. The procedure was repeated at 6 weeks. After 6 weeks, hilar cholangiocarcinoma occurred in the treatment group and model group. Then, the treatment group was injected with GW4064 intraperitoneally at a dose of 50 mg/kg/day. One week after injection, the rats in the four groups were euthanized. Pathological examination confirmed that tumours had formed, and hilar bile duct tissues were taken from the four groups. FXR, Bsep, Ntcp and NF-κB expression in the hilar bile duct was detected by real-time polymerase chain reaction (RT–PCR) and immunohistochemistry. After three weeks, the rats in the treatment group and model group ate less, and their weight was significantly reduced. Six weeks later, hilar cholangiocarcinoma was detected in the treatment group and model group. After treatment with GW4064, the ratios of FXR/GAPDH mRNA, Bsep/GAPDH mRNA, Ntcp/GAPDH mRNA and NF-κBp65/GAPDH mRNA were significantly different among the four groups. Under a light microscope, FXR protein reacted with anti-FXR antibody, Bsep protein reacted with anti-Bsep antibody, Ntcp protein reacted with anti-Ntcp antibody and NF-κBp65 protein reacted with anti-NF-κBp65 antibody, and they showed granular expression. Every pathological section included 4,800 cells, and there were different numbers of positive cells in each group. FXR expression in the hilar cholangiocarcinoma of rats was significantly lower than that in normal hilar bile duct tissues. GW4064 increased the expression of FXR in tumour tissues. These findings suggest that FXR may be a new therapeutic target and that GW4064 may be helpful in the treatment of hilar cholangiocarcinoma.

## Introduction

Malignant tumours of the extrahepatic bile duct may occur in the upper (hilar cholangiocarcinoma), middle and lower segments of the extrahepatic bile duct. Among these tumours, hilar cholangiocarcinoma has the highest incidence and is the most difficult to treat. According to the statistical data of various medical institutions, the treatment methods dominated by surgery and supplemented by chemotherapy, radiotherapy and interventional therapy have progressed with the development of technology. Although these advancements have solved the problems of biliary obstruction and rapid tumour growth for some patients, the five-year survival rate of patients has not significantly improved. If the patient has severe cardiopulmonary dysfunction or liver and kidney dysfunction and is not suitable for the above treatment methods, the treatment is more difficult, and the patient's quality of life is significantly reduced. Therefore, there is an urgent need for new treatments^1–3^.

Gene therapy is a new method in tumour therapy that has been developed in recent years for use in the treatment of some malignant tumours of the digestive system and respiratory system. The combination of gene therapy and other treatment methods improves the quality of life and prolongs the survival time of patients. However, few target genes have been found in hilar cholangiocarcinoma. Therefore, researchers are trying to identify genes related to the pathogenesis of hilar cholangiocarcinoma as therapeutic targets. What gives us a glimmer of hope is that in previous experiments, we found that the expression of FXR was decreased in hilar cholangiocarcinoma. An agonist of FXR (GW4064) has been found. Therefore, we designed an experiment to study whether the size of hilar cholangiocarcinoma and the expression of FXR changed after the use of FXR agonists. We hope to find a new target and a new drug for the treatment of hilar cholangiocarcinoma^4–6^.

## Materials and methods

### Statement

The study was carried out in compliance with the ARRIVE guidelines.

### Rats

EightY Wistar rats (male, 180 ± 5 g) were provided by the Animal Experimental Center of Southwest Medical University. They were randomly divided into four groups (treatment group, model group, control group and sham operation group, n = 20 each). Before the study, the rats were healthy and had no other diseases.

### Experimental methods

The materials used include the following: microsyringes (Tianjin Chenhang Keyuan Technology Development Co., Ltd), pentobarbital sodium (Beijing Younikang Biotechnology Co., Ltd.), the QBC939 human cholangiocarcinoma cell line (Shanghai Tongpai Biotechnology Co., Ltd.), GW4064(Zhejiang Haiqiang Chemical Co., Ltd), DMEM (Shenzhen kangchuyuan Co., Ltd), anti-FXR antibody (Chemicon USA), anti-Bsep antibody (Chemicon USA), anti-Ntcp antibody (Chemicon USA), anti-NFκBp65 antibody(Shanghai Xinyu Biotechnology Co., Ltd.), Real-time PCR kits (SR1100) and ABI7500 real-time PCR detection system (Singapore). Tissue sections were prepared using ethanol, xylene, paraffin and a sectioning mechanism. The sense and antisense primers used to detect FXR mRNA levels were as follows: 5′-CCTCATTGTCTCCCCGACTTA-3′ and 5′-ACTTGTGACG AAAGATCTCCG-3′. The sense and antisense primers used to detect Bsep mRNA were as follows: 5′-CCCTCAACTGATGGGGGCTCCAGT-3′ and 5′-CCCATGTCTGACTCAG TGATTCTT-3′. The sense and antisense primers used to detect Ntcp mRNA were as follows: 5′-GATGGAGGTGCACAACGTAT-3′ and 5′-CTGTCTCAGTTCATGGCTCC-3′. The sense and antisense primers used to detect NF-κBp65mRNA levels were as follows: 5′-TAGCCTCAGGGTACTCCATCA-3′ and 5′-GGGAAGGAACGCTGTCAGAG-3′. The sense and antisense primers used to detect GAPDH mRNA levels were as follows: 5′-GATGGTGGGTATGGGTCAGAA-3′ and 5′-CTAATGACGGGACCGAGGATC-3′. The 2^−∆∆Ct^ method was used to normalize the data. The diameter of the needle tip of the microsyringe was 40 μm and was connected to a 100 µL syringe through a rubber tube.

### Establishment of the animal model

All Wistar rats were fed a standard diet. The culture environment of QBC939 human cholangiocarcinoma cells was set at 37 °C and 5% saturated humidity, and the culture medium was DMEM. The cells with poor growth will be eliminated, and the cells with good growth will be preserved. Extract well-growing cells, prepare cell suspension (a concentration of 1 × 10^6^ cells/mL), and inoculate Wistar rats. The treatment group, model group were anaesthetized with 1.5% pentobarbital sodium and 0.2 ml/100 g intraperitoneal injection. After the abdomen was disinfected, it was cut along the midline. Then, the injection needle connected to the microinjector was inserted into the hilar bile duct and 100 µl of tumor cell suspension was injected at a uniform speed. After careful hemostasis, the abdominal structures were sutured in turn to complete the operation. A modified tail suspension test (TST) was used to evaluate the mental state and physical activity of rats every day. At 4 weeks, one rat in the treatment group and model group was euthanized respectively, and the changes in the shape and size of the hilar bile duct were carefully recorded. The procedure was repeated at 6 weeks. After 6 weeks, hilar cholangiocarcinoma occurred in the treatment group and model group. Then the treatment group was injected with GW4064 intraperitoneally at a dose of 50 mg/kg/day. One week after injection, the rats in four groups were euthanized. Pathological examination confirmed the formation of tumours, and hilar bile duct tissues were taken from the four groups. RT–PCR was used to detect the expression levels of FXR mRNA, Bsep mRNA, Ntcp mRNA and NF-κBp65 mRNA (RNA was extracted from hilar cholangiocarcinoma and normal hilar bile duct), and GAPDH expression level served as an internal control. Immunohistochemistry was used to analyse the expression of FXR, Bsep, Ntcp and NF-κBp65 protein. Under the light microscope, FXR protein reacted with anti-FXR antibody, Bsep protein reacted with anti-Bsep antibody, Ntcp protein reacted with anti-Ntcp antibody and NF-κBp65 protein reacted with anti-NFκBp65 antibody, they showed granular expression. Each pathological section was divided into four areas: upper left, lower left, upper right and lower right. 1200 cells were observed in each area. Tissues with a positive cell rate greater than 10% are considered positive, and tissues with a positive cell rate less than 10% are considered negative.

### Statistical analysis

Data are presented as the mean ± SD. SPSS 22.0 statistical software was used for data analysis. The *t* test was used to judge the differences between two groups. The χ^2^ test was used to evaluate immunohistochemistry data, and *P* < 0.05 was used to indicate statistically significant differences.

### Ethics approval and informed consent

The study protocol was approved by the Ethics Committee of the affiliated hospital, Southwest Medical University, Luzhou, Sichuan Province, China. Number: swmu20220049. Animal welfare guidelines abided by China Laboratory Animal Welfare Law and Animal management regulations, Number: GB/T 35892‐20181.

## Results

### General condition and tumor size change of rats

**Figure 1:**
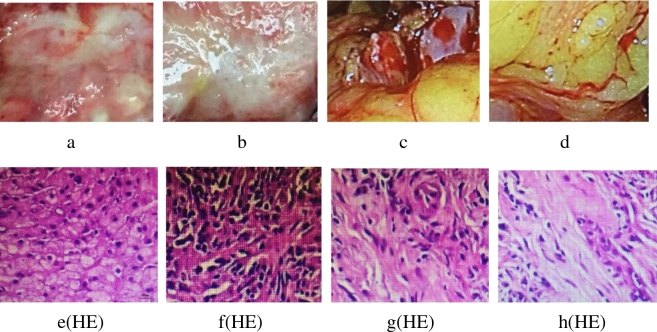
Pathological examination. (**a**) Hilar cholangiocarcinoma in treatment group. (**b**) Hilar cholangiocarcinoma in model group. (**c**) Hilar bile duct in control group. (**d**) Hilar bile duct in sham operation group. (**e**) Hilar cholangiocarcinoma tissues in treatment group. HE stain (magnification 100×). (**f**) Hilar cholangiocarcinoma tissues in model group. HE stain (magnification 100×). (**g**) Hilar bile duct tissues in control group. HE stain (magnification 100×). (**h**) Hilar bile duct tissues in sham operation group. HE stain (magnification 100×). Through pathologic examination, we detected hilar cholangiocarcinoma in 17 rats (85%) in the treatment group and 16 rats (80%) in the model group after seven weeks. But in control group and sham operation group the inflammation and edema of hilar bile duct are mild, and a small amount of inflammatory cells and macrophages infiltrate between tissues, the mitotic image of tumor nuclei was common. After GW4064 treatment, although the size of the tumor did not change significantly, but some necrosis began to appear in the tumor tissues.

From the third week, the food intake of rats in the treatment group and the model group gradually decreased, and their weight decreased compared with the control group and the sham operation group (Tables 1, 2).Two rats in the treatment group died and three rats in the model group died after 6 weeks. There were no deaths in the other two groups. Through pathologic examination, we detected hilar cholangiocarcinoma in 17 rats (85%) in the treatment group and 16 rats (80%) in the model group after 7 weeks (Fig. 1). The changes in liver function in the four groups are shown in Table 3. The changes of tumor size in the treatment group and model group are shown in Table 4.

**Table 1:** Daily food-intake (g).

Weeks	Control group (n = 20)	Sham operation group (n = 20)	Model group (n = 20)	Treatment group (n = 20)
3	19.15 ± 0.07	19.23 ± 0.09	19.18 ± 0.07	19.21 ± 0.06
4	23.58 ± 0.08	23.67 ± 0.11	21.26 ± 0.09	21.59 ± 0.08
5	28.01 ± 0.14*	27.39 ± 0.12^^^	18.75 ± 0.08	17.98 ± 0.07
6	29.14 ± 0.13^#^	29.59 ± 0.14^&^	17.03 ± 0.05	16.89 ± 0.06
7	31.23 ± 0.12^$^	30.78 ± 0.13^@^	16.29 ± 0.06	17.11 ± 0.05

*P < 0.05 compared with the model group.

^^^*P* < 0.05 compared with the model group.

^#^*P* < 0.05 compared with the model group.

^&^*P* < 0.05 compared with the model group.

^$^*P* < 0.05 compared with the model group.

^@^*P* < 0.05 compared with the model group. After 4 weeks the rats in model group and treatment group ate less, due to the formation and progression of hilar cholangiocarcinoma, bile secretion is blocked and dyspepsia occurs, their food intake is significantly reduced compared with the other two groups. However, after GW4064 treatment, the food intake of the treatment group increased slightly in the seventh week.

**Table 2:** Body mass (g).

Week	Control group (n = 20)	Sham operation group(n = 20)	Model group (n = 20)	Treatment group (n = 20)
3	205 ± 2.6	206 ± 3.2	205 ± 2.8	206 ± 3.3
4	219 ± 3.7	220 ± 4.3	221 ± 3.9	221 ± 4.2
5	235 ± 4.1*	236 ± 4.5^^^	203 ± 3.1	202 ± 2.9
6	251 ± 4.7^#^	252 ± 5.0^&^	194 ± 2.1	195 ± 2.2
7	260 ± 5.2^$^	263 ± 5.1^@^	181 ± 1.6	199 ± 1.8^α^

*P < 0.05 compared with the model group.

^^^*P* < 0.05 compared with the model group.

^#^*P* < 0.05 compared with the model group.

^&^*P* < 0.05 compared with the model group.

^$^*P* < 0.05 compared with the model group.

^@^*P* < 0.05 compared with the model group.

^α^*P* < 0.05 compared with the model group. After four weeks the rats in model group and treatment group ate less, due to the formation and progression of hilar cholangiocarcinoma, bile secretion is blocked and dyspepsia occurs, their food intake is significantly reduced compared with the other two groups, so the weight is also reduced accordingly. However, after GW4064 treatment, the food intake of the treatment group increased slightly in the seventh week, so the weight also increases accordingly.

**Table 3:** Changes of liver function (the seventh week).

Related indicators	Control group (n = 20)	Sham operation group (n = 20)	Model group (n = 17)	Treatment group (n = 18)
(ALT)/(U/L)	61.85 ± 2.05^$^	62.14 ± 2.23^$$^	158.37 ± 4.87	122.59 ± 3.16^$$$^
(AST)/(U/L)	63.62 ± 2.14^&^	64.36 ± 2.38^&&^	161.67 ± 4.29	128.71 ± 3.09^&&&^
(TC)/(mmol/L)	2.53 ± 0.12*	2.55 ± 0.14**	6.61 ± 0.17	5.24 ± 0.16
(TBA)/(μmol/L)	1.75 ± 0.08^#^	1.74 ± 0.07^##^	5.27 ± 0.13	4.62 ± 0.14
(TBIL)/(μmol/L)	3.07 ± 0.05^@^	3.21 ± 0.06^@@^	5.76 ± 0.08	4.23 ± 0.07^@@@^
(DBIL)/(μmol/L)	1.73 ± 0.07^^^	1.71 ± 0.05^^^^	3.92 ± 0.07	2.88 ± 0.13^^^^^

In control group, ^$^*P* < 0.05, ^&^*P* < 0.05, *P < 0.05, ^#^*P* < 0.05, ^@^*P* < 0.05, ^^^*P* < 0.05 compared with the model group. In sham operation group, ^$$^*P* < 0.05, ^&&^*P* < 0.05, **P < 0.05, ^##^*P* < 0.05, ^@@^P < 0.05, ^^P < 0.05 compared with the model group. In treatment group, ^$$$^*P* < 0.05, ^&&&^*P* < 0.05, ^@@@^P < 0.05, ^^^P < 0.05 compared with the model group. The levels of total cholesterol, total bilirubin, direct bilirubin, alanine aminotransfease and aspartate transaminase, in model group and treatment group were higher compared with the other two groups. However, in the seventh week, after GW4064 treatment, the damaged liver function of rats in the treatment group was gradually recovered.

*ALT* alanine aminotransfease, *AST* aspartate transaminase, *TC* total cholesterol, *TBA* total bile acids, *TBIL* Total bilirubin, *DBIL* direct bilirubin.

**Table 4:** Tumor size (the seventh week).

Week	Model group (mm)	Treatment group (mm)
7	17.1 ± 0.3	16.8 ± 0.2

The tumor size in the treatment group was slightly smaller than that in the model group, although there is no significant statistical difference between the two groups at this time, the growth of tumor has been inhibited.

### Analysis of FXR, Bsep, Ntcp and NF-κBp65 expression

**Figure 2:**
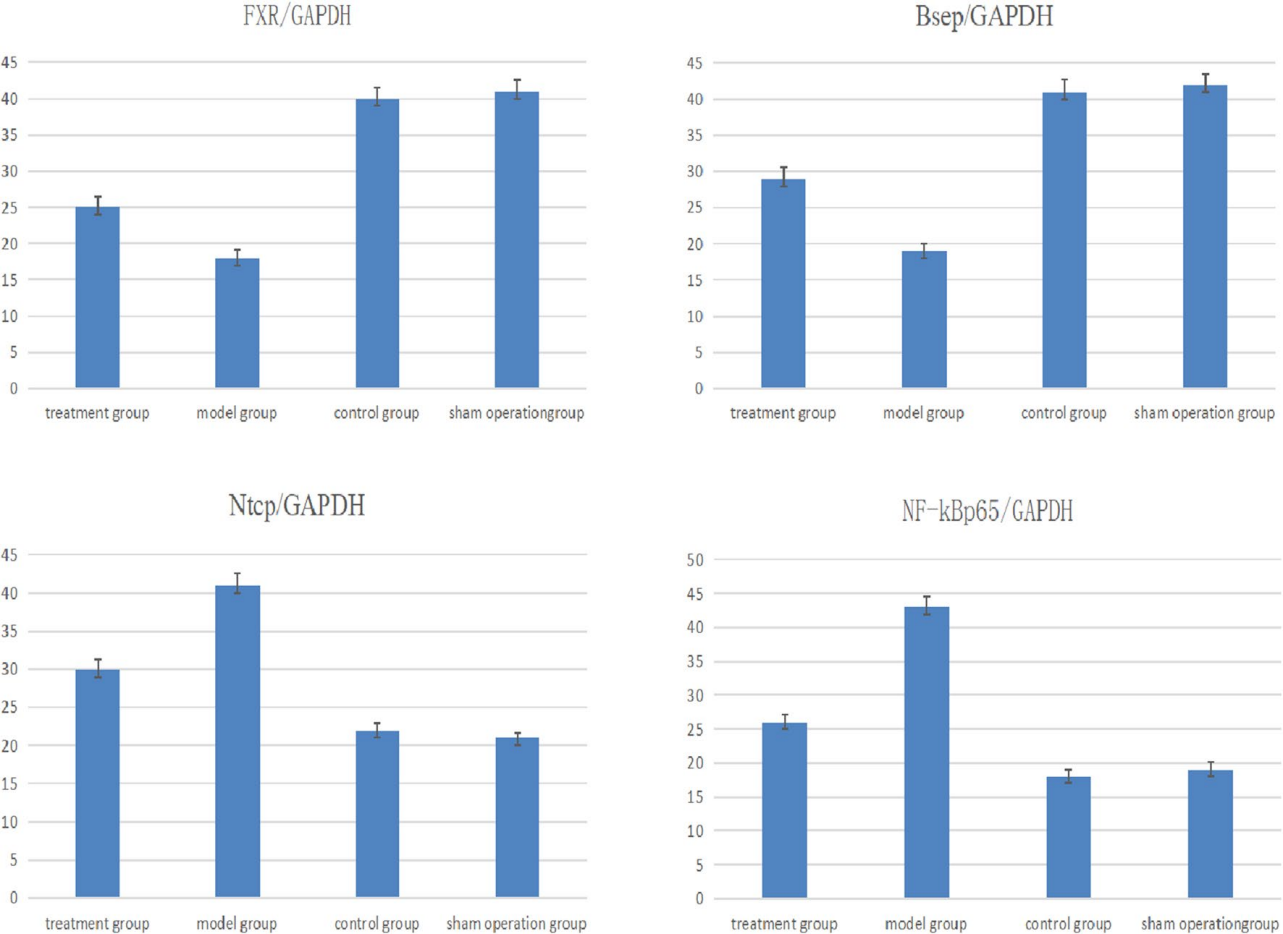
Analysis of FXR mRNA, Bsep mRNA, Ntcp mRNA and NF-κBp65 mRNA expression by RT-PCR. Through RT–PCR, we found that in the treatment group, model group, control group and sham operation group, there were significant difference among the four groups in the ratios of FXR/GAPDH, FXR/GAPDH, FXR/GAPDH and NF-κBp65/GAPDH. In the model group, FXR and Bsep decreased significantly compared with the control group, while Ntcp and NF-κBp65 increased significantly. After GW4064 treatment, FXR and Bsep in the treatment group increased compared with the model group, while Ntcp and NF-κBp65 continued to decrease, inflammation was controlled, cholestasis was improved, and the situation was developing in a better direction.

Through RT–PCR, we found that in the treatment group, model group, control group and sham operation group, the FXR/GAPDH ratios were 25 ± 1.4, 18 ± 1.2, 40 ± 1.5 and 41 ± 1.6, respectively. After eighteen cycles, there was a significant difference among the four groups (between the model group and treatment group, *t* = 3.046, *P* < 0.05; between the model group and control group, *t* = 3.356, *P* < 0.05; between the model group and sham operation group, *t* = 3.297, *P* < 0.05). The Bsep/GAPDH ratios were 29 ± 1.6, 19 ± 1.1, 41 ± 1.7 and 42 ± 1.5, respectively. After eighteen cycles, there was a significant difference among the four groups (between the model group and treatment group, *t* = 3.174, *P* < 0.05; between the model group and control group, *t* = 3.679, *P* < 0.05; between the model group and sham operation group, *t* = 3.712, *P* < 0.05). The Ntcp/GAPDH ratios were 30 ± 1.3, 41 ± 1.6, 22 ± 0.9 and 21 ± 0.7, respectively. After eighteen cycles, there was a significant difference among the four groups (between the model group and treatment group, *t* = 3.214, *P* < 0.05; between the model group and control group, *t* = 3.558, *P* < 0.05; between the model group and sham operation group, *t* = 3.562, *P* < 0.05). The NF-κBp65/GAPDH ratios were 26 ± 1.2, 43 ± 1.5, 18 ± 1.0 and 19 ± 1.1, respectively. After eighteen cycles, there was a significant difference among the four groups (between the model group and treatment group, *t* = 3.107, *P* < 0.05; between the model group and control group, *t* = 3.426, *P* < 0.05; between the model group and sham operation group, *t* = 3.437, *P* < 0.05) (Fig. 2).

**Figure 3:**
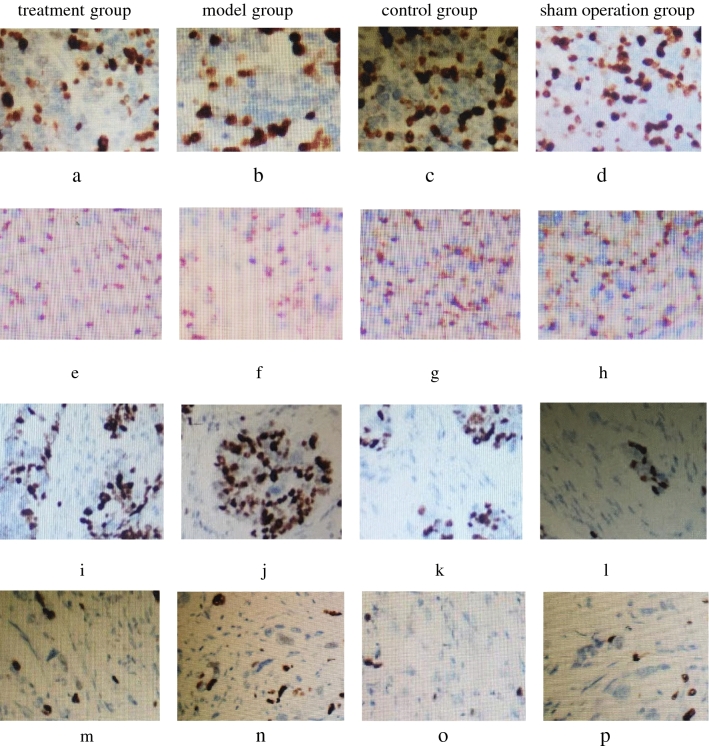
Analysis of FXR, Bsep, Ntcp and NF-κBp65 expression by immunohistochemical assay. From photo (**a**) to photo (**d**): FXR expression in treatment group, model group, control group and sham operation group (magnification 200×). From photo (**e**) to photo (**h**): Bsep expression in treatment group, model group, control group and sham operation group (magnification 200×). From photo (**i**) to photo (**l**): Ntcp expression in treatment group, model group, control group and sham operation group (magnification 200×). From photo (**m**) to photo (**p**): NF-κBp65 expression in treatment group, model group, control group and sham operation group (magnification 200×). FXR protein reacted with the anti-FXR antibody, Bsep protein reacted with anti-Bsep antibody, Ntcp protein reacted with anti-Ntcp antibody and NF-κBp65 protein reacted with anti-NF-κBp65 antibody. Every pathological section included 4800 cells. FXR protein expression, Bsep protein expression, Ntcp protein expression and NF-κBp65 protein expression were significant difference among the four groups.

FXR protein reacted with anti-FXR antibody, Bsep protein reacted with anti-Bsep antibody, Ntcp protein reacted with anti-Ntcp antibody and NF-κBp65 protein reacted with anti-NFκBp65 antibody, and they are shown in Fig. 3. Every pathological section included 4800 cells. FXR protein is expressed as follows: a total of 2517 positive cells (52.4%) were in the treatment group, 1739 positive cells (36.2%) were in the model group, 3361 positive cells (70.0%) were in the control group, 3417 positive cells (71.1%) were in the sham operation group, and there was significant difference among the four groups (χ^2^ = 35.12, *P* < 0.05, between the model group and treatment group, χ^2^ = 37.38, *P* < 0.05, between model group and control group; χ^2^ = 38.24, *P* < 0.05, between model group and sham operation group.). Bsep protein is expressed as follows: a total of 2736 positive cells (57.0%) were in the treatment group, 1674 positive cells (34.8%) were in the model group, 3275 positive cells (68.2%) were in the control group, 3354 positive cells (69.9%) were in the sham operation group, and there was significant difference among the four groups (χ^2^ = 33.51, *P* < 0.05, between the model group and treatment group, χ^2^ = 36.72, *P* < 0.05, between model group and control group; χ^2^ = 37.01, *P* < 0.05, between model group and sham operation group.). Ntcp protein is expressed as follows: a total of 2374 positive cells (49.5%) were in the treatment group, 3319 positive cells (69.1%) were in the model group, 1683 positive cells (35.1%) were in the control group, 1637 positive cells (34.1%) were in the sham operation group, and there was significant difference among the four groups (χ^2^ = 33.86, *P* < 0.05, between the model group and treatment group, χ^2^ = 35.47, *P* < 0.05, between model group and control group; χ^2^ = 36.13, *P* < 0.05, between model group and sham operation group.). NF-κBp65 protein is expressed as follows: a total of 2336 positive cells (48.7%) were in the treatment group, 3862 positive cells (80.5%) were in the model group, 1028 positive cells (21.4%) were in the control group, 1179 positive cells (24.6%) were in the sham operation group, and there was significant difference among the four groups (χ^2^ = 34.24, *P* < 0.05, between the model group and treatment group, χ^2^ = 36.59, *P* < 0.05, between model group and control group; χ^2^ = 37.15, *P* < 0.05, between model group and sham operation group.).

## Discussion

There is a high probability that malignant tumours of the biliary tract occur in the extrahepatic biliary tract, and hilar cholangiocarcinoma is the most common tumour in the extrahepatic biliary tract. Because it may invade the hepatic artery and portal vein in the early stage, surgical resection is extremely difficult. However, it is difficult to obtain obvious results with conservative treatment. Despite long-term basic research and clinical reports, we know that the factors related to hilar cholangiocarcinoma include clonorchiasis, bile duct stones, and biliary ascaris, and we have found some tumour characteristics in our continuous exploration. At present, the changes in tumour gene expression and the regulatory mechanism of specific signal transduction pathways related to hilar cholangiocarcinoma are not completely clear^7–10^. For example, hilar cholangiocarcinoma may be caused by the joint action of multiple signalling pathways caused by different genes, but whether these factors regulate and promote each other has not been completely clarified. This may be the reason for the lack of effective treatment. Usually, early tumours can be resolved by surgery, and the prognosis is good. However, if the tumour infiltrates the surrounding blood vessels or metastases, surgery cannot solve the main problem. The effects of chemotherapy, interventional therapy and traditional Chinese medicine are limited. In the existing treatment methods, the recurrence and metastasis rate of hilar cholangiocarcinoma is high, and the survival rate is low. Therefore, more effective treatment is the first problem to be solved. Gene therapy is a new method that has been developed in recent years. At present, it has been used to treat malignant tumours in some systems, but no effective gene has been found to be a therapeutic target in hilar cholangiocarcinoma. Therefore, researchers are trying to identify the genes associated with hilar cholangiocarcinoma^11–13^.

FXR is usually expressed on the surface of hepatocytes and bile ducts. It has many functions, especially important in several aspects. First, it affects tumor growth through specific signal transduction pathways. When FXR is highly expressed, tumor growth is inhibited. On the contrary, when FXR is reduced, tumor growth may continue. Therefore, we found that the expression of FXR in tumor cells of hilar cholangiocarcinoma was significantly lower than that in normal tissues^14^. Second, it controls the excretion and reabsorption of bile acids by regulating the target gene of bile salt export pump (BSEP, regulation of bile acid secretion) and sodium taurocholate transporter (NTCP, regulation of bile acid reabsorption), so as to maintain the stability of bile acid concentration^15^, avoid excessive accumulation of bile acids in the bile duct, lead to the occurrence of stones and cholangitis, cause repeated destruction and proliferation of bile duct cells, the emergence of heterogenous cells, and promote the further development of tumors. Therefore, in the experimental results we found that in the model group, FXR decreased significantly, so it caused Bsep to decrease and Ntcp to increase. On the one hand, bilirubin increased and bile acids accumulated in the bile duct. On the other hand, glutamic pyruvic transaminase and glutamic oxaloacetic transaminase increased, and liver function was significantly affected. Third, it regulates NF-κB signaling pathway affects the development of inflammation^16,17^, when NF-κB rises, this signal pathway is obviously activated, which leads to more significant inflammatory reaction, promotes the edema, destruction and repeated proliferation of cells, and increases the probability of cells with malignant phenotype, thus further promoting the progress of tumors. Therefore, in the experimental results, we found that in the model group, NF-κB increased significantly with obvious inflammatory cell infiltration, but it not emerge in normal tissues^18–21^. Previous studies have shown that the expression level of FXR in hepatocellular carcinoma was significantly decreased, and the incidence of liver tumours in FXR gene knockout mice was 100%. FXR deletion can activate the Wnt/β-catenin and RalA-GTP carcinogenic pathways, leading to hepatocarcinogenesis. In patients with hepatitis B, FXR deletion can promote the occurrence of liver cancer by reducing the transcriptional activity of FXR-KNG1 signalling. In vitro cell experiments we have demonstrated that FXR is expressed in hepatocellular carcinoma cells and that FXR agonists can inhibit the proliferation of hepatoma cells^22–25^.

FXR agonists have been found to play a role in the treatment of a few tumours. Therefore, we considered whether FXR agonists could be used to treat hilar cholangiocarcinoma^26–28^. GW4064 is a highly effective and selective nonsteroidal FXR agonist. In CV-1 cells transfected with mouse and human FXR expression vectors and identified reporter genes, the EC50 values were 80 and 90, respectively. GW4064 at concentrations up to 1 μm also had no activity on other nuclear receptors, including retinoic acid receptors. Therefore, when we used GW4064 in the treatment group, the corresponding treatment effect began to appear. First, although the size of the tumour did not change significantly, the FXR content in the tumour tissue began to rise, and some necrosis began to appear in the tumour tissue. Second, in the treatment group, Bsep increased and Ntcp decreased, liver function damage was reduced, alanine aminotransferase and glutamic oxaloacetic aminotransferase were reduced, the bilirubin content was reduced, and the degree of cholestasis was improved. Again, as NF-κB decreases, the inflammatory reaction decreases, and the oedema gradually subsides. Therefore, the inflammatory cell infiltration in the tumour tissue and surrounding tissues decreases, the cell swelling decreases, and the endoplasmic reticulum gradually returns to the normal state. Therefore, we preliminarily determined the expression of FXR in different tissues and the therapeutic effect of GW4064^29–31^.

At present, there are few drugs that can treat cholangiocarcinoma, especially hilar cholangiocarcinoma, and they mainly focus on inhibiting tumour angiogenesis. However, drugs that can directly act on tumour bodies have not been clearly found, and statistics show that existing drugs have different side effects in the treatment process. First, this kind of medicine needs to be taken for a long time, which is difficult for many patients to adhere to. Second, gastrointestinal bleeding, skin rash, abnormal liver function, nausea and vomiting and other reactions may occur during the course of treatment, and some patients have serious reactions. Third, the scope of this kind of drug treatment is limited to some tumours with specific pathological classifications, but it is ineffective for others. Furthermore, once the drug is stopped during the course of treatment, the tumour will quickly metastasize. Drugs in animal research and clinical experiments do not directly act on tumour cells. Therefore, after FXR expression detection, we tried to use GW4064 to treat hilar cholangiocarcinoma. Other FXR agonists, such as ASC42 and WAY-362450, have been used to treat primary biliary cholangitis (PBC), primary sclerosing cholangitis (PSC) and liver fibrosis, and these diseases are the possible causes of hilar cholangiocarcinoma. Clinical trials of these drugs at different stages have gradually begun, and a large amount of data have been obtained. They may lay a foundation for the treatment of hilar cholangiocarcinoma^32,33^. The current basic research may improve our understanding of gene expression patterns and the related signalling pathways in hilar cholangiocarcinoma, and these findings could be verified in clinical experiments. However, there are still many problems that need further observation and understanding. First, FXR is not limited to tumour cells. Will FXR agonists affect normal tissues while being used to treat tumours? Currently, few data on this are available. Second, can GW4064 be used locally? If it was directly injected into the bile duct, could it enhance the treatment effects? In the coming days, there will be more exploration and more detailed research to evaluate the reliability and stability of new treatments. From the existing statistical results and survival analysis data, the development of targeted therapy has improved in this field^34,35^. In this study, we achieved several objectives. First, we successfully established a rat model of hilar cholangiocarcinoma and then observed the expression changes of FXR, Bsep, Ntcp and NF-κB in normal hilar bile duct tissues and tumour tissues. Finally, we preliminarily identified the changes in FXR, Bsep, Ntcp and NF-κB after treatment with GW4064. This study will help us explore new therapeutic targets for hilar cholangiocarcinoma according to the changes in FXR and explore the role of FXR agonists, such as GW4064, in the treatment of hilar cholangiocarcinoma.

## Data Availability

The authors confirm that the data supporting the findings of this study are available within the article.
